# Long-term outcomes of endovascular thrombectomy vs. medical care in patients with large ischemic stroke: a systematic review and meta-analysis of randomized controlled trials

**DOI:** 10.3389/fneur.2026.1776595

**Published:** 2026-05-13

**Authors:** Jiayu You, Hang Zhou, Qianshuo Liu, Xingqiang Li

**Affiliations:** 1 Department of Neurology and Neuroscience, Shenyang First People’s Hospital, Shenyang Brain Institute, Shenyang, Liaoning, China; 2 Department of Neurology, The Fourth Affiliated Hospital of China Medical University, Shenyang, Liaoning, China

**Keywords:** Alberta Stroke Program Early CT score, endovascular thrombectomy, large ischemic infarct, large vessel occlusion, medical care, randomized controlled trial

## Abstract

**Background:**

Several randomized controlled trials (RCTs) have shown endovascular thrombectomy (EVT) to be superior to standard medical care (MC) for anterior circulation large vessel occlusion (LVO)-related large ischemic infarcts at the 90-day follow-up, but long-term (≥90 days) safety and effectiveness evidence is lacking.

**Objectives:**

This meta-analysis used high-quality RCTs (1-year follow-up) to assess EVT’s clinical benefits in this patient group and compare short- vs. long-term prognostic changes.

**Search methods:**

Literature searches were conducted in PubMed/MEDLINE, Scopus, and Web of Science from their inception to 28 September 2025 for RCTs comparing EVT and MC in acute anterior circulation ischemic stroke (AIS) with large ischemic infarcts. Study quality was evaluated using the Cochrane risk of bias tool.

**Selection criteria:**

These included RCTs enrolled patients with confirmed anterior circulation LVO and low Alberta Stroke Program Early CT Score [ASPECTS] ≤ 5, compared EVT and MC, and reported long-term outcome data.

**Data collection and analysis:**

A meta-analysis of long-term functional/safety outcomes was performed; subgroup analyses were performed based on onset time, ASPECTS, and imaging screening methods, along with leave-one-out sensitivity analysis.

**Results:**

Baseline characteristics were balanced between the groups, and all included RCTs were high-quality. At long-term follow-up (>90 days to 12 months), EVT significantly improved functional excellence [modified Rankin Scale 0–1; risk ratio (RR) = 3.84, 95% confidence interval (CI) = 2.35–6.28; *p* < 0.001], functional independence (modified Rankin Scale 0–2; RR = 3.13, 95%CI = 2.01–4.86; *p* < 0.001), and independent ambulation (modified Rankin Scale 0–3; RR = 2.01, 95%CI = 1.52–2.67; *p* < 0.001) in patients with anterior circulation large ischemic infarcts; mortality was not significantly different between groups (RR = 0.90, 95%CI = 0.78–1.05; *p* = 0.19). EVT also reduced the risk of death or dependency (modified Rankin Scale 4–6; RR = 0.78, 95%CI = 0.73–0.84; *p* < 0.001). Long-term follow-up revealed more significant prognostic improvements with EVT compared with short-term follow-up.

**Conclusion:**

In AIS patients with anterior circulation LVO-related large ischemic infarcts, EVT plus MC yielded statistically significant long-term functional improvements compared with MC alone. EVT’s benefits were amplified with longer follow-up and were more pronounced in patients with shorter onset time and smaller infarct volume.

**Systmatic review registraion:**

https://www.crd.york.ac.uk/prospero/display_record.php?ID=CRD420251144703, CRD420251144703.

## Introduction

For acute ischemic stroke (AIS) due to anterior circulation large vessel occlusion (LVO), endovascular thrombectomy (EVT) is widely recognized as the gold standard therapy based on multiple randomized controlled trials (RCTs) and international guidelines (1). However, the landmark RCTs validating EVT’s efficacy used strict enrollment criteria, typically excluding patients with large ischemic infarcts—defined by low Alberta Stroke Program Early CT Score (ASPECTS) or large ischemic core volumes on perfusion imaging (2–8).

Patients with low ASPECTS were excluded from earlier EVT trials primarily due to two concerns: diminished efficacy (owing to the belief that early ischemic changes indicate unsalvageable tissue) and elevated hemorrhagic risk (as infarcted tissue is prone to hemorrhagic transformation post-reperfusion) (9). Therefore, EVT’s efficacy and safety in large-core cerebral infarction remained unclear.

Six recent RCTs (RESCUE-Japan LIMIT, ANGEL-ASPECTS, SELECT2, TESLA, TENSION, and LASTE) (10–15) directly compared EVT and standard MC in this patient group (primarily focusing on short-term outcomes), challenging the prior assumption that these patients would not benefit from EVT and would be at excessive risk of hemorrhage. Key mechanisms underlying EVT’s benefits include reduced infarct volume [demonstrated in LASTE (10) and SELECT2 (11)]; even when infarct volume did not drop significantly [TENSION (13), ANGEL-ASPECTS (14), and RESCUE-Japan LIMIT (15)], EVT may act via ischemic injury gradient utilization and partial tissue salvage. Additionally, mediation analyses have shown that reduced cerebral edema—measured by midline shift and net water uptake—contributes more to functional recovery than penumbra salvage in these patients (16, 17).

A meta-analysis of the six RCTs (10–15) confirmed that EVT improves 90-day functional outcomes (modified Rankin Scale, mRS) in patients with 6-h onset, ASPECTS 3–5, and intracranial internal carotid artery (ICA)/proximal middle cerebral artery (M1) occlusion (18). Several RCTs included follow-up for more than 90 days [LASTE: 180 days (11) and SELECT2/TESLA/TENSION/ANGEL-ASPECTS: 12 months (19–22)], offering insight into the long-term sustainability of EVT’s benefits and the absence of delayed risks, thereby addressing gaps in short-term data (11, 19–22). Based on these RCTs, the 2026 American Heart Association/American Stroke Association (AHA/ASA) guidelines emphasize refined patient selection for reperfusion therapies and updated prognostic stratification in AIS, particularly for patients with low ASPECTS (23). Amid accumulating evidence and ongoing debate, a meta-analysis is crucial to synthesize data, clarify EVT’s long-term efficacy and safety, and compare short- vs. long-term prognostic changes.

## Materials and methods

### Standardized reporting and registration

We adhered to the Cochrane systematic review methodological framework and the Preferred Reporting Items for Systematic reviews and Meta-Analyses (PRISMA) reporting standards (24), with the protocol prospectively registered in the International Prospective Register of Systematic Reviews (PROSPERO) (CRD420251144703).

### Search strategy

We searched PubMed/MEDLINE, Scopus, and Web of Science from their inception to 28 September 2025, with no language restrictions. Tailored search strategies combining terms for “endovascular thrombectomy” and “large stroke” (keywords/MeSH terms) were used for each database; reference lists of published trials were also manually screened for comprehensiveness.

### Study selection

We included records that met the following eligibility criteria:

### Population

We included studies that enrolled adult patients (≥18 years) with AIS and large infarct cores due to acute anterior circulation LVO, as identified on baseline neuroimaging (defined as an ASPECTS ≤ 5 by any modality or an infarct core volume ≥ 50 mL). Subgroup analyses were pre-specified according to infarct core size and imaging criteria. Studies involving posterior circulation LVO patients were excluded. *In vitro*, animal, and postmortem studies were excluded.

### Intervention

We included studies comparing EVT with standard MC, with no restrictions on symptom onset-to-treatment time. We also pre-specified assessments of various endovascular adjunctive procedures to thrombectomy (e.g., stenting and angioplasty).

### Outcomes

The primary outcomes were functional independence (mRS score: 0–2) and all-cause mortality, assessed within 1-year follow-up post-randomization. The secondary outcomes included functional excellence (mRS score: 0–1), independent ambulation (mRS score: 0–3), death or dependency (mRS score: 4–6), and survival (mRS score: 0–5). The short-term outcomes were measured at 90 days post-randomization, and long-term follow-up was defined as >90 days: SELECT2, TESLA, ANGEL-ASPECTS, and TENSION defined this at 1 year, whereas LASTE defined this at 180 days.

### Study designs

We included only RCTs, given their high quality of evidence and suitability for quantitative evaluation, and excluded other study designs (e.g., observational studies, editorials, commentaries, guidelines, reviews, meta-analyses, conference abstracts, and news pieces).

### Screening

Reviewers first completed calibration with piloted, standardized screening forms to ensure consistency. Citations were then independently screened and verified by two-reviewer teams (ZH and LQS). Full texts of potentially eligible citations were retrieved after initial screening, with each screened text assessed independently by one reviewer and verified by another (ZH and YJY); disagreements were resolved by consulting the corresponding author (LXQ).

### Data extraction

Two authors (ZH and LQS) independently extracted data using a pre-standardized Excel sheet, with a third author verifying all data for accuracy; discrepancies were resolved via team discussion or third-party adjudication. Extracted variables included study features (e.g., country and design), patient characteristics (e.g., sample size, age, National Institutes of Health stroke scale (NIHSS) score, and vessel occlusion), intervention details (e.g., IV thrombolysis use), and outcomes (mRS scores, mortality, intracranial hemorrhage, and procedural complications).

### Risk of bias assessment

The methodological quality of the included studies was assessed using the Cochrane risk of bias (ROB2) tool, a standardized instrument for evaluating the risk of bias in RCTs, which covers seven key domains: random sequence generation, allocation concealment, blinding of participants/personnel, blinding of outcome assessors, incomplete outcome data handling, selective reporting, and other potential bias sources.

### Data synthesis and analysis

Data synthesis involved narrative descriptions and tabular summaries, with the initial meta-analysis performed using Review Manager 5. For patient-important outcomes reported in ≥2 studies, dichotomous outcomes were pooled using the Mantel–Haenszel method to calculate risk ratios (RRs) with 95%CIs, while continuous outcomes were pooled as standardized mean differences (SMDs). Funnel plots were used to assess publication bias via asymmetry. Cochran’s *Q* test and *I*^2^ evaluated statistical heterogeneity: a fixed-effects model was used for non-significant heterogeneity (*p* > 0.1, *I*^2^ < 40%), and a random-effects model was used for significant heterogeneity (*p* < 0.1, *I*^2^ > 40%). Comprehensive synthesis, including subgroup and sensitivity analyses, was conducted using Stata/MP 17.

### Sensitivity analyses

Sensitivity analyses were performed to assess the robustness of the findings through model alteration and leave-one-out meta-analysis (omitting one trial at a time). Trials with high heterogeneity or high risk of bias were excluded to rerun analyses and achieve homogeneity; however, this approach was not applicable to meta-analyses including only two studies (since only one study would remain after exclusion). Statistical significance was defined as a *p*-value of <0.05.

## Results

**Figure 1:**
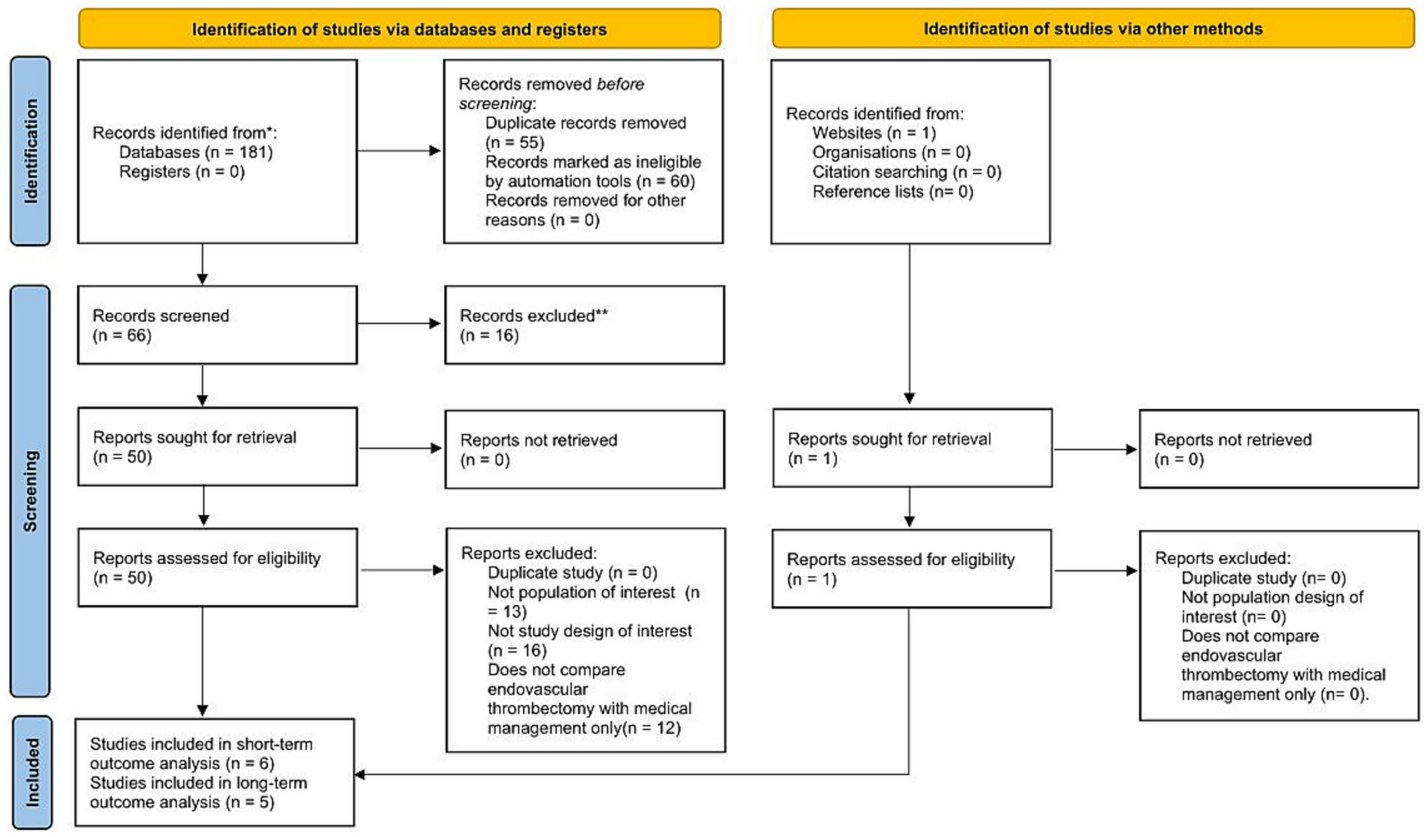
PRISMA flowchart for study selection.

A systematic search identified 181 records; after removing duplicates, screening titles and abstracts, and assessing full-text, 5 RCTs with long-term follow-up (>90 days to 12 months) comparing EVT and MC in large ischemic infarcts were included, along with 6 short-term (≤90 days) RCTs for functional outcome subgroup analysis. The study selection process is shown in the PRISMA flowchart (Figure 1).

### Study characteristics and comparison of baseline study characteristics

We evaluated the baseline characteristics of participants in each RCT to identify parameter disparities, with RCT characteristics for EVT vs. MC in anterior circulation large ischemic infarct patients outlined in Table 1. Overall, baseline demographics were well-balanced across trials, with only coronary artery disease showing a statistically significant difference (Table 2); atrial fibrillation was the only baseline variable exhibiting significant heterogeneity.

**Table 1:** Study characteristics of the included studies.

Study	LASTE (10)	SELECT2 (11)	TESLA (12)	TENSION (13)	ANGEL (14)	RESCUE (15)
Location	Europe, United States	North America and Australia	United States	Europe and Canada	China	Japan
Enrollment time	April 2019 to March 2022	September 2019 to September 2022	16 July 2019 to 17 October 2022	17 July 2018 to 21 February 2023	2 October 2020 to 18 May 2022	16 July 2019 to 17 October 2022
Inclusion criteria						
Age (years)	≥18	18–85	18–85	≥18	18–80	≥18
Pre-stroke mRS	0 or 1	0 or 1	0 or 1	0 to 2	0 or 1	0 or 1
Site of occlusion	ICA, M1, or M2	ICA or M1	ICA or M1	ICA or M1	ICA or M1	ICA or M1
Time window	Up to 7 h (or MRI FLAIR negative)	Up to 24 h	Up to 24 h	Up to 12 h	Up to 24 h	Up to 6 h (or MRI FLAIR negative)
ASPECTS criteria	CT or MR ASPECTS 0–5	CT ASPECTS 3–5	CT ASPECTS 2–5	CT or MR ASPECTS 3–5	CT ASPECTS 3–5	CT or MR ASPECTS 3–5
Additional imaging criteria	ASPECTS must be 4 or 5 for age ≥80 years	Core volume ≥50 mL also qualified. Patients with established infarcts were excluded	–	–	Core volume 70–100 mL also qualified	–
Short-term (90 days) RCTs						
Patients	324	352	300	253	455	203
EVT/MC	159/165	178/174	152/148	125/128	230/225	101/102
EVT age, y	73 (66–79)	66 (58–75)	66 (54–74)	73 (65–81)	68 (61–73)	76.0 (10.0)
MC age, y	74 (65–80)	67 (58–75)	67.5 (57.5–73.5)	74 (64–80)	67 (59–73)	75.7 (10.2)
EVT male	82	107	76	69	135	55
MC male	88	100	84	61	144	58
IVT in EVT	55	37	31	49	66	27
IVT in MC	58	30	30	44	63	29
Baseline NIH in EVT	21 (18–24)	19 (15–23)	19 (15–23)	19 (16–22)	16 (13–20)	22 (18–26)
Baseline NIH in MC	21 (18–24)	19 (15–22)	18 (14.5–21)	18 (15–22)	15 (12–19)	22 (17–26)
ASPECT of EVT	NA	4 (3–5)	4 (3–5)	NA	3 (3–4)	3 (3–4)
ASPECT of MC	NA	4 (4–5)	4 (3–5)	NA	3 (3–4)	4 (3–4)
mRS in EVT	4 (3–6)	4 (3–6)	5 (3–6)	4 (3–6)	4 (2–5)	
mRS in MC	6 (4–6)	5 (4–6)	5 (4–6)	6 (4–6)	4 (3–5)	
mRS 0–2 in EVT	21/158	36/177	22/151	21/124	69	14
mRS 0–2 in MC	8/164	12/171	13/146	3/122	26	8
Mortality in EVT	57	68/177	53/150	49/122	50	18
Mortality in MC	91	71/171	49/147	63/123	45	24
Long-term (180 days–1 year) RCTs						NA
Patients	319	329	277	244	425	–
EVT/MC	159/165	178/174	144/133	125/128	214/211	–
mRS in EVT	4 (3–6)	5 (3–6)	3.654 ± 0.217	5 (3–6)	3 (2–6)	–
mRS in MC	6 (4–6)	6 (4–6)	2.776 ± 0.172	6 (4–6)	4 (3–6)	–
ASPECT 3–5 in EVT	73	NA	NA	103	185	–
ASPECT 3–5 in MC	70	NA	NA	99	182	–
mRS 0–2 in EVT	29	40	32	27	65	–
mRS 0–2 in MC	8	9	8	7	36	–
mRS 0–3 in EVT	58	63	49	42	107	–
mRS 0–3 in MC	21	29	21	20	75	–
Mortality in EVT	64	77	64	58	67	–
Mortality in MC	92	83	60	70	56	–

EVT, endovascular thrombectomy; MC, medical care; mRS, modified Rankin Scale; IVT, intravenous thrombolysis; NA, not applicable; ICA, internal carotid arteries; M1 and M2, middle cerebral artery; NIH, National Institutes of Health Stroke Scale; ASPECTS, Alberta Stroke Program Early CT Score; FLAIR, Fluid-Attenuated Inversion Recovery.

**Table 2:** Comparison of baseline characteristics between the two groups.

Variable	EVT (820)	MC (811)	Effect size [95%CI]	*p*	*T* ^2^	*I* ^2^
Patient age, mean [standard deviation (SD)], y	68.66 (11.50)	69.43 (11.69)	SMD = −0.42 [−1.5–0.66]	0.45	0	0%
Females	367/820 (44.8%)	355/811 (43.8%)	RR = 1.02 [0.92–1.13]	0.69	0	0%
Past medical history						
Hypertension	465/676	464/678	RR = 1.01 [0.94–1.08]	0.88	0	0%
Congestive heart failure	40/303	35/302	RR = 1.14 [0.75–1.74]	0.55	0	0%
Coronary artery disease	106/462	78/467	RR = 1.37 [1.06–1.79]	0.02	0	0%
Atrial fibrillation	162/676	173/678	RR = 0.93 [0.70–1.23]	0.61	0.05	56%^*^
Diabetes	179/676	184/678	RR = 0.98 [0.82–1.16]	0.81	0	0%
Vessel occlusion location						
ICA	269/676	254/678	RR = 1.06 [0.93–1.21]	0.38	0	0%
M1	395/676	412/678	RR = 0.96 [0.88–1.05]	0.38	0	0%
M2	10/676	10/678	RR = 0.96 [0.88–1.05]	0.98	0	0%
IV tPA usage	192/676	184/678	RR = 1.05 [0.89–1.25]	0.55	0	0%
NIHSS at admission, mean (SD)	18.52(5.43)	18.05(5.52)	SMD = −0.49 [−0.04–1.02]	0.07	0.04	12%
ASPECTS at admission, mean (SD)	3.22(1.43)	3.2(1.27)	SMD = 0.01 [−0.08–0.11]	0.79	0	0%
General anesthesia	203/462	NA	NA	NA	NA	NA
Crossover between the groups rate	8/462	5/467	NA	NA	NA	NA
Transfer to thrombectomy-capable center	382/676	381/678	RR = 1.01 [0.92–1.10]	0.91	0	0%

EVT, endovascular thrombectomy; MC, medical care; RR, risk ratio; CI, confidence interval; ICA, internal carotid arteries; M1 and M2, middle cerebral artery; IV tPA, intravenous tissue plasminogen activator; NIHSS, National Institutes of Health Stroke Scale; ASPECTS, Alberta Stroke Program Early CT Score.

^*^Analysis was conducted using a random-effect model.

### Quality assessment

**Figure 2:**
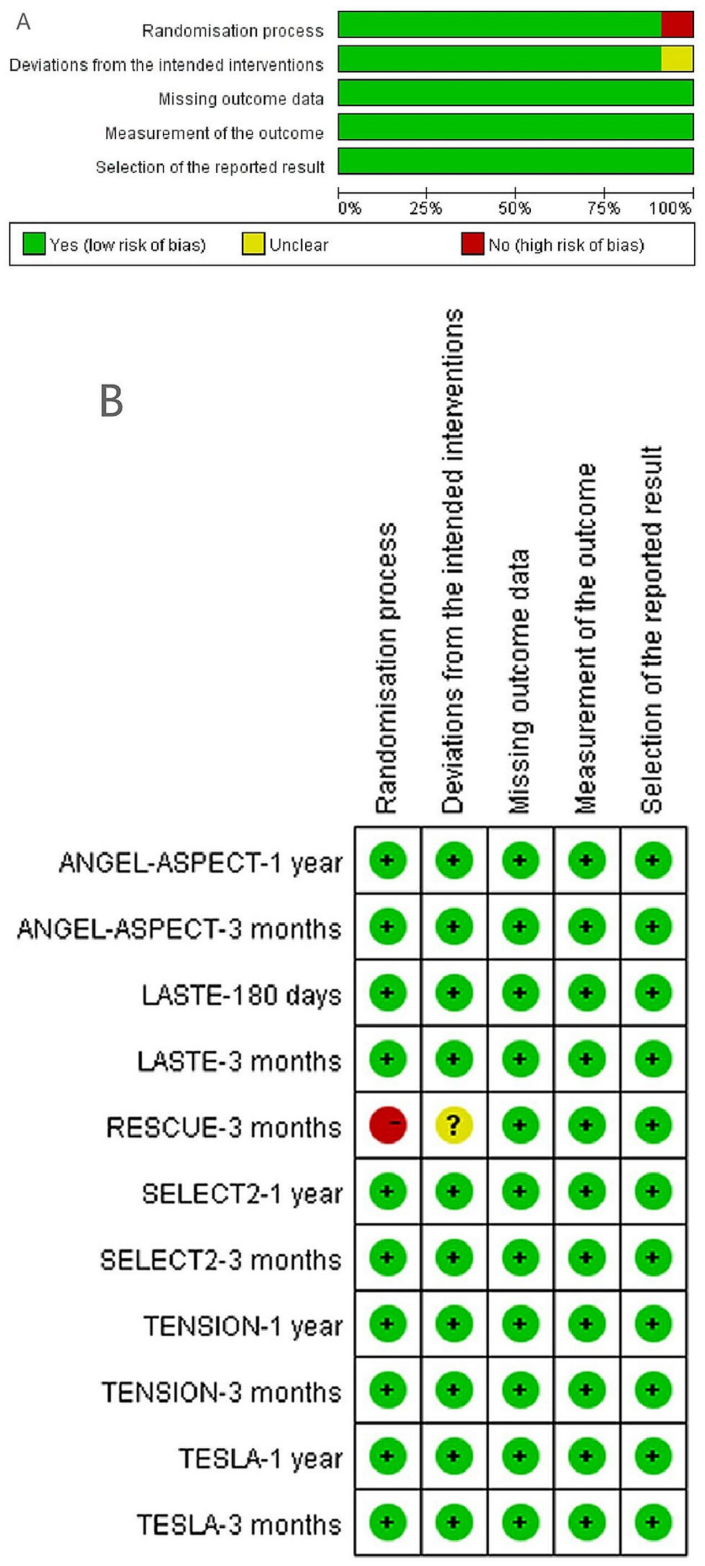
Risk of bias assessment. **(A)** Revised Cochrane risk of bias tool (ROB2). **(B)** Risk of bias summary.

All included studies demonstrated a low risk of bias across the five core ROB2 domains (Figure 2A). The majority of trials exhibited high methodological quality, minimizing design and execution bias, and supporting the reliability of outcomes.

Conversely, the 3-month RESCUE trial had two limitations (Figure 2B): a high risk of bias in the randomization process and an unclear risk of bias due to deviation from interventions. Sensitivity analyses were conducted by rerunning the primary meta-analysis after excluding the 3-month RESCUE data to verify result stability.

### Outcomes of EVT compared with MC alone

#### Functional excellence (mRS score: 0–1)

All included studies reported functional excellence (mRS score: 0–1).

**Figure 3:**
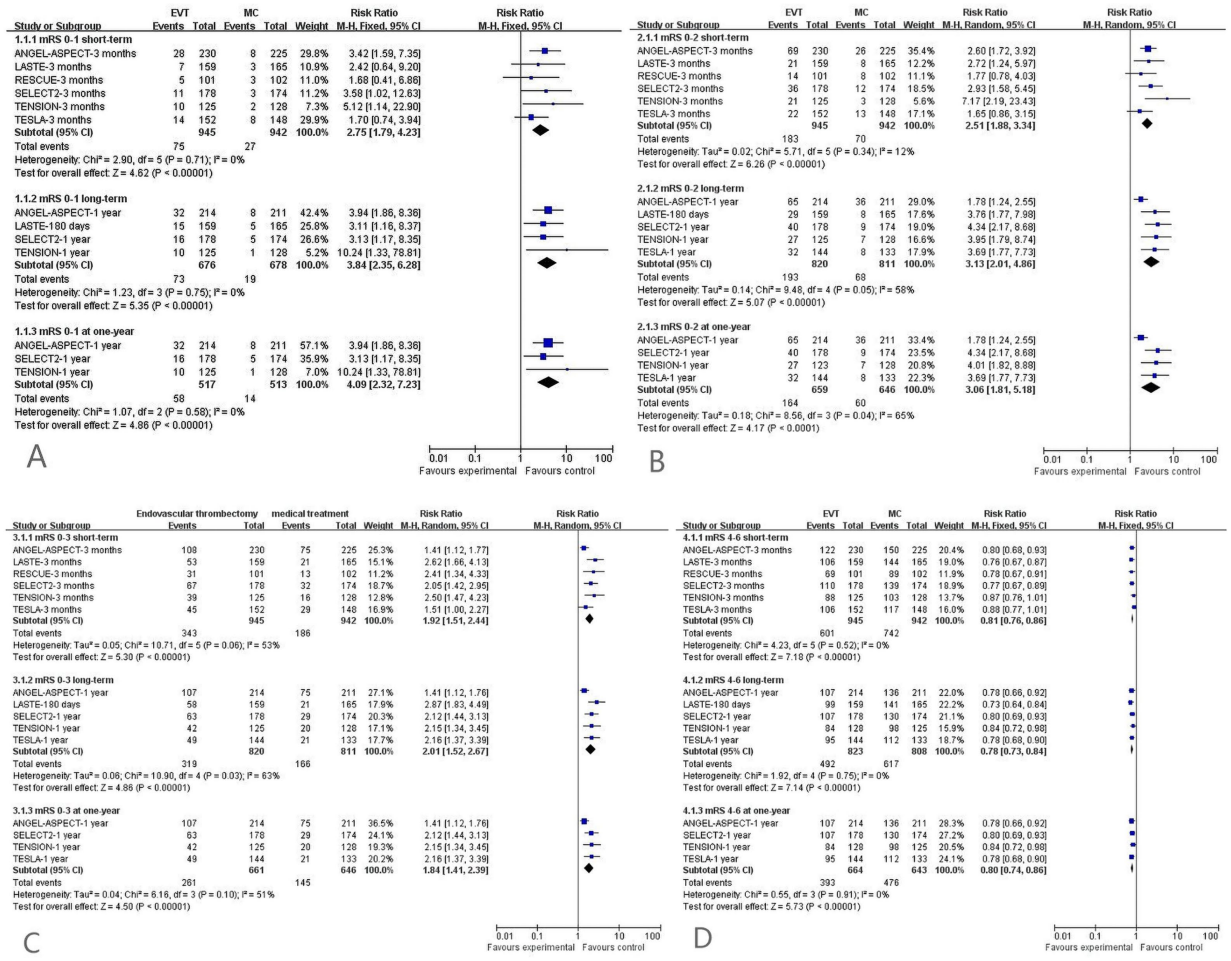
**(A)** Meta-analysis forest plot for functional excellence (mRS score: 0–1); **(B)** meta-analysis forest plot for functional independence (mRS score: 0–2); **(C)** meta-analysis forest plot for independent ambulation (mRS score: 0–3); **(D)** meta-analysis forest plot for death or dependency (mRS score: 4–6).

- Short-term (≤90 days) RCTs: Data from 1,887 patients (6 RCTs) showed a significantly higher rate of functional excellence in the EVT group than in the MC group [75/945 (7.9%) vs. 27/942 (2.9%); RR = 2.75, 95%CI = 1.79–4.23; *p* < 0.001], with low heterogeneity (*I*^2^ = 0%, *p* = 0.71) (Figure 3A).
- Long-term (>90 days to 12 months) RCTs: Data from 1,354 patients (4 RCTs) showed a significantly higher rate in the EVT group than in the MC group [73/676 (10.8%) vs. 19/678 (2.8%); RR = 3.84, 95%CI = 2.35–6.28; *p* < 0.001], with low heterogeneity (*I*^2^ = 0%, *p* = 0.75) (Figure 3A).
- Long-term (12 months) RCTs: Data from 1,030 patients (3 RCTs) showed a significantly higher rate in the EVT group than in the MC group [58/517 (11.2%) vs. 14/513 (2.7%); RR = 4.09, 95%CI = 2.32–7.23; *p* < 0.001], with low heterogeneity (*I*^2^ = 0%, *p* = 0.58) (Figure 3A).

#### Functional independence (mRS score: 0–2)

All included studies reported functional independence (mRS score: 0–2).

- Short-term (≤90 days) RCTs: Data from 1,887 patients (6 RCTs) showed that the EVT group had a higher rate of functional independence than the MC group [183/945 (19.4%) vs. 70/942 (7.4%); RR = 2.51, 95%CI = 1.88–3.34; *p* < 0.001], with low heterogeneity (*I*^2^ = 12%, *p* = 0.34) (Figure 3B).
- Long-term (>90 days to 12 months) RCTs: Data from 1,631 patients (5 RCTs) showed that the EVT group had a higher rate than the MC group [193/820 (23.5%) vs. 68/811 (8.4%); RR = 3.13, 95%CI = 2.01–4.86; *p* < 0.001], with moderate heterogeneity (*I*^2^ = 58%, *p* = 0.05) (Figure 3B).
- Long-term (12 months) RCTs: Data from 1,305 patients (4 RCTs) showed that the EVT group had a significantly higher rate in than the MC group [164/659 (24.9%) vs. 60/646 (9.3%); RR = 3.06, 95%CI = 1.81–5.18; *p* < 0.001], with moderate heterogeneity (*I*^2^ = 65%, *p* = 0.04) (Figure 3B).

#### Independent ambulation (mRS score: 0–3)

All included studies reported independent ambulation (mRS score: 0–3).

- Short-term (≤90 days) RCTs: Data from 1,887 patients (6 RCTs) showed that the EVT group had a significantly higher independent ambulation rate than the MC group [343/945 (36.3%) vs. 186/942 (19.7%); RR = 1.92, 95%CI = 1.51–2.44; *p* < 0.001], with moderate heterogeneity (*I*^2^ = 53%, *p* = 0.06) (Figure 3C).
- Long-term (>90 days to 12 months) RCTs: Data from 1,631 patients (5 RCTs) showed that the EVT group had a significantly higher independent ambulation rate than the MC group [319/820 (38.9%) vs. 166/811 (20.5%); RR = 2.01, 95%CI = 1.52–2.67; *p* < 0.001], with moderate heterogeneity (*I*^2^ = 63%, *p* = 0.03) (Figure 3C).
- Long-term (12 months) RCTs: Data from 1,307 patients (4 RCTs) showed that the EVT group had a significantly higher independent ambulation rate than the MC group [261/661 (39.5%) vs. 145/646 (22.4%); RR = 1.84, 95%CI = 1.41–2.39; *p* < 0.001], with moderate heterogeneity (*I*^2^ = 51%, *p* = 0.10) (Figure 3C).

#### Death or dependency (mRS score: 4–6)

All included studies reported death or dependency (mRS score: 4–6).

- Short-term (≤90 days) RCTs: Data from 1,887 patients (6 RCTs) showed that the EVT group had a significantly lower death or dependency rate than the MC group [601/945 (63.6%) vs. 742/942 (78.8%); RR = 0.81, 95%CI = 0.76–0.86; *p* < 0.001], with low heterogeneity (*I*^2^ = 0%, *p* = 0.52) (Figure 3D).
- Long-term (>90 days to 12 months) RCTs: Data from 1,631 patients (5 RCTs) showed that the EVT group had a significantly lower death or dependency rate than the MC group [492/823 (59.8%) vs. 617/808 (76.4%); RR = 0.78, 95%CI = 0.73–0.84; *p* < 0.001], with low heterogeneity (*I*^2^ = 0%, *p* = 0.75) (Figure 3D).
- Long-term (12 months) RCTs: Data from 1,307 patients (4 RCTs) showed that the EVT group had a significantly lower death or dependency rate than the MC group [393/664 (59.2%) vs. 476/643 (74.0%); RR = 0.80, 95%CI = 0.74–0.86; *p* < 0.001], with low heterogeneity (*I*^2^ = 0%, *p* = 0.91) (Figure 3D).

### Mortality

All included studies reported mortality, defined as an mRS score of 6.

**Figure 4:**
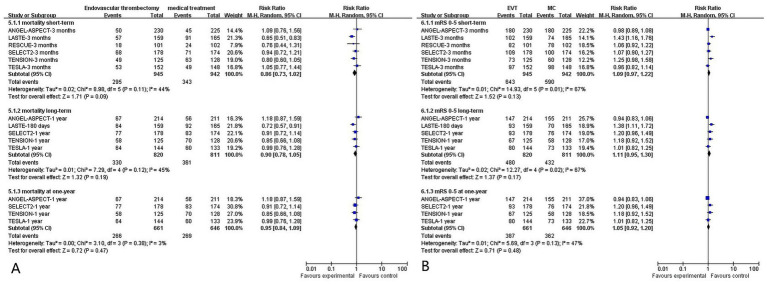
**(A)** Meta-analysis forest plot for mortality; **(B)** meta-analysis forest plot for overall survival (mRS 0–5).

- Short-term (≤90 days) RCTs: Data from 1,887 patients (6 RCTs) showed that the EVT group had lower mortality than the MC group [295/945 (31.2%) vs. 343/942 (36.4%); RR = 0.86, 95%CI = 0.73–1.02; *p* = 0.09], with low heterogeneity (*I*^2^ = 44%, *p* = 0.11) (Figure 4A).
- Long-term (>90 days to 12 months) RCTs: Data from 1,631 patients (5 RCTs) showed no significant difference in mortality between the EVT and MC groups [330/820 (40.2%) vs. 361/811 (44.5%); RR = 0.90, 95%CI = 0.78–1.05; *p* = 0.19], with low heterogeneity (*I*^2^ = 45%, *p* = 0.12) (Figure 4A).
- Long-term (12 months) RCTs: Data from 1,307 patients (4 RCTs) showed no significant difference in mortality between the EVT and MC groups [266/661 (40.2%) vs. 269/646 (41.6%); RR = 0.97, 95%CI = 0.85–1.10; *p* = 0.6], with low heterogeneity (*I*^2^ = 3%, *p* = 0.38) (Figure 4A).

#### Overall survival (mRS score: 0–5)

All included studies reported overall survival, defined as an mRS score of 0–5.

- Short-term (90 days) RCTs: Among 1,887 patients from 6 RCTs, overall survival did not differ significantly between the EVT and MC groups [68.0% (643/945) vs. 62.6% (590/942); RR = 1.09, 95%CI = 0.97–1.22; *p* = 0.13], with moderate heterogeneity (*I*^2^ = 67%, *p* = 0.01) (Figure 4B).
- Long-term (>90 days to 12 months) RCTs: For 1,631 patients, no significant between-group difference was observed in overall survival [58.5% (480/821) vs. 53.7% (432/805); RR = 1.11, 95%CI = 0.95–1.30; *p* = 0.17], with moderate heterogeneity (*I*^2^ = 67%, *p* = 0.02) (Figure 4B).
- Long-term (12 months) RCTs: For 1,307 patients, no significant between-group difference was observed in overall survival [58.5% (387/661) vs. 56.0% (362/646); RR = 1.05, 95%CI = 0.92–1.20; *p* = 0.48], with moderate heterogeneity (*I*^2^ = 47%, p = 0.13) (Figure 4B).

### Subgroup analysis

Subgroup analyses of functional outcomes are presented in Table 3, and forest plots are shown in Supplementary Figures 5–19. Pooled analyses showed a significant 1-year mRS distribution shift favoring EVT over MC in patients with ASPECTS of 3–5 (pooled OR = 1.43, 95%CI = 1.03–2.00, *p* = 0.03, Supplementary Figure 18); however, there was no such shift in those with ASPECTS of 0–2 or onset ≥6 h post-last known well (Supplementary Figures 17, 19). Regardless of perfusion imaging-based selection, EVT was associated with higher long-term favorable functional outcome rates (mRS score: 0–2/0–3) than MC (Supplementary Figures 5, 6, 9, 10). For patients with onset ≤12/24 h, EVT also yielded higher long-term favorable outcome proportions (mRS score: 0–2/0–3) (Supplementary Figures 7, 8, 11, 12); notably, EVT patients with onset ≤12 h had significantly lower mortality than the MC group (Supplementary Figure 15).

**Table 3:** Subgroup analysis on functional outcomes.

Basis	Subgroups	Trials	Effect size [95%CI]	Subgroup difference	*I* ^2^
Functional independence	Long-term follow-up^a^	5	RR = 3.13 [2.01–4.86]	*p* = 0.05	58%
	Short-term follow-up^a^	6	RR = 2.51 [1.88–3.34]	*p* = 0.34	12%
	Perfusion imaging^b^	2	RR = 2.65 [1.10–6.39]	*p* = 0.02	81%
	No-perfusion imaging	3	RR = 3.81 [2.46–5.91]	*p* = 0.99	0%
	Presented ≤12 h^c^	2	RR = 3.88 [2.25–6.70]	*p* = 0.91	0%
	Presented ≤24 h^c^	3	RR = 2.88 [1.53–5.42]	*p* = 0.03	71%
Independent ambulation	Long-term follow-up	5	RR = 2.01 [1.52–2.67]	*p* = 0.03	63%
	Short-term follow-up	6	RR = 1.92 [1.51–2.44]	*p* = 0.06	53%
	Perfusion imaging	2	RR = 1.68 [1.12–2.51]	*p* = 0.07	70%
	No-perfusion imaging	3	RR = 2.39 [1.83–3.11]	*p* = 0.60	0%
	Presented ≤12 h	2	RR = 2.52 [1.82–3.48]	*p* = 0.39	0%
	Presented ≤24 h	3	RR = 1.78 [1.30–2.45]	*p* = 0.08	60%
Mortality	Long-term follow-up	5	RR = 0.91 [0.81–1.01]	*p* = 0.12	45%
	Short-term follow-up	6	RR = 0.86 [0.76–0.97]	*p* = 0.11	44%
	Perfusion imaging	2	RR = 1.02 [0.79–1.31]	*p* = 0.17	47%
	No-perfusion imaging	3	RR = 0.84 [0.72–0.96]	*p* = 0.22	34%
	Presented ≤12 h	2	RR = 0.78 [0.66–0.92]	*p* = 0.35	0%
	Presented ≤24 h	3	RR = 1.01 [0.87–1.17]	*p* = 0.39	0%
1-year outcomes	mRS = 0–2	4	RR = 3.06[1.81–5.18]	*p* = 0.04	65%
	mRS = 0–3	4	RR = 1.84 [1.41–2.39]	*p* = 0.10	51%
	Mortality	4	RR = 0.97 [0.85–1.10]	*p* = 0.38	3%
1-year mRS shift	ASPECTS = 0–2	3	Pooled OR = 0.88 [0.60–1.31]	*p* = 0.46	0%
	ASPECTS = 3–5	3	Pooled OR = 1.43 [1.03–2.00]	*p* = 0.19	39%
	Presented ≥6 h	3	Pooled OR = 1.56 [0.77–3.18]	*p* = 0.0009	86%

mRS, modified Rankin Scale; ASPECTS, Alberta Stroke Program Early CT Score; RR, risk ratio; CI, confidence interval.

a Long-term outcomes are defined as outcomes reported within 1-year follow-up, and short-term outcomes are defined as outcomes reported up to 90 days follow-up.

b RCT(s) utilized perfusion imaging for patient selection.

c The presented ≤12 h is defined as trials that included patients in ≤12 h from last-known-well to randomization (LASTE 8 ≤ 7 h and TENSION 7 ≤ 12 h), and presented ≤24 h is defined as trials that included patients in the whole window.

### Sensitivity analyses

Pooled result stability and sensitivity were assessed via statistical model modification: switching from fixed- to random-effects models still showed higher favorable outcome rates with EVT than MC, confirming the robustness of the meta-analysis results (Supplementary Figures 20–24).

Leave-one-out sensitivity analysis for long-term functional independence (>90 days to 12 months) evaluated the impact of individual studies on EVT-MC comparisons. Overall, the combined RRs ranged from 2.89 (95%CI = 1.78–4.70) to 3.94 (95%CI = 2.72–5.71), with all *p*-values of <0.001, demonstrating that the findings are robust and unaffected by any single study.

## Discussion

In this RCT meta-analysis, we compared the efficacy and safety of EVT vs. MC alone in anterior circulation LVO patients with large ischemic infarcts. Key strengths include a focus on long-term outcomes and a comparative analysis against the standard 90-day follow-up for primary outcomes in the majority of trials.

Traditionally, acute large ischemic infarct patients were excluded from the majority of thrombectomy studies due to concerns over sICH and limited functional recovery (7, 25). However, recent trials (e.g., RESCUE-Japan LIMIT and ANGEL-ASPECTS) confirmed EVT’s clinical utility in this population (ASPECTS 0–5) (14, 15), with SELECT2 and LASTE showing improved 90-day functional independence and quality of life (10, 11). Published 1-year RCT follow-up results (e.g., SELECT2 (19), TESLA (20), TENSION (21), and ANGEL-ASPECTS (22)) demonstrate sustained EVT benefits with greater improvements in long-term functional recovery, while LASTE (10) demonstrates benefits up to 6 months. These findings challenge the prior view that thrombectomy offers little benefit and carries excessive risk for large ischemic core patients.

We acknowledge the valuable contributions of the four published meta-analyses (26–29) to this field, and our study builds on their foundation while presenting distinct and important innovations. As the first analysis with literature retrieval updated to 28 September 2025, our study includes the latest trial data and updated long-term follow-up information not fully covered by previous meta-analyses (29); for the first time, we systematically and quantitatively compared the short-term (6 studies) and long-term (5 studies) prognostic trajectories of EVT, instead of only focusing on a single time point as in prior studies (26–28). Different from previous meta-analyses (26, 27), we strictly restricted our population to LVO patients with ASPECTS ≤5, a clinically challenging large-core infarct subgroup with high homogeneity, which renders our conclusions more targeted for clinical practice. Furthermore, our study is the first to quantify the long-term benefits of EVT on functional excellence (mRS score: 0–1), to quantify a stricter functional endpoint, and to establish a complete evaluation system by assessing independent ambulation (mRS score: 0–3) and death or dependency (mRS score: 4–6). Most importantly, we quantitatively demonstrate that the prognostic benefits of EVT are significantly amplified with prolonged follow-up, a key novel finding that extends beyond the qualitative descriptions provided in previous meta-analyses.

This RCT meta-analysis shows that EVT significantly improves key long-term outcomes [functional excellence (mRS score: 0–1), independence (mRS score: 0–2), and ambulation (mRS score: 0–3)] in patients with large ischemic cores (ASPECTS 0–5). Although all-cause mortality and overall survival did not differ between groups, EVT tripled the likelihood of 1-year functional independence (*p* < 0.05) and reduced the risk of death or dependency by 22% (statistically significant), supporting EVT as a viable option for this once-ineligible population. While EVT benefits were evident at 90 days, functional recovery continued to 1 year [1-year mRS score: 0–2: 3.06 (1.81–5.18); 3-month mRS score: 0–2: 2.51 (1.88–3.34)], highlighting the value of extended follow-up. These findings emphasize the need for longer-term assessments in future EVT trials, as 90-day evaluations may underestimate the full therapeutic benefits and fail to assess delayed complications that are critical for guiding clinical decisions.

Our results align with the 2026 AHA/ASA guideline, which emphasizes individualized prognostication and shared decision-making for patients with low ASPECTS. Notably, EVT showed no significant 1-year mRS improvement in patients with ASPECTS of 0–2 or symptom onset >6 h within the 24-h window, unlike the favorable outcomes in patients with ASPECTS of 3–5. Extensive irreversible cerebral damage (ASPECTS: 0–2, core volume: ≥100 mL) may explain this, as even successful recanalization fails to yield meaningful functional recovery. While EVT remains effective up to 24 h, treatment beyond 6 h may reduce benefits in patients with ASPECTS of 0–2, highlighting the time-sensitive nature of intervention when large core size and delayed onset act as limiting barriers. In contrast, patients with ASPECTS of 3–5 (core volume: 50–100 mL) have sufficient salvageable tissue to benefit from EVT, leading to better outcomes than MC and the aforementioned subgroups. These findings underscore the critical importance of patient selection—considering ASPECTS stratification, onset time, and core volume <150 mL—to maximize thrombectomy benefits. The observed 1-year mRS shift patterns provide real-world evidence to support the guideline’s updated recommendations regarding the potential for functional recovery in this subgroup, informing clinical counseling and treatment planning.

In conclusion, our meta-analysis suggests that EVT confers substantial long-term efficacy in improving functional outcomes among patients with acute extensive ischemic strokes (ASPECTS 0–5), particularly in ASPECTS of 3–5 and onset less than 6 h. Notably, the benefits of EVT extend beyond the 90-day follow-up, with sustained recovery observed up to 1-year follow-up.

### Limitations

This study has notable strengths but also several limitations. First, long-term outcomes showed inter-study statistical heterogeneity likely due to methodological differences. Second, five of the six included trials enrolled only ICA/M1 occlusion patients, leaving the safety of EVT in distal occlusions (e.g., distal M1, M2) unclear. Third, while ASPECTS is a practical tool for estimating infarct size, it does not accurately quantify ischemic core volume; variability in imaging modalities [magnetic resonance imaging (MRI) vs. computed tomography (CT)] across trials may further contribute to selection variability. Fourth, posterior circulation LVO was excluded due to the extremely limited number of eligible RCTs and insufficient long-term follow-up data to support a robust meta-analysis. Finally, early trial termination for efficacy may lead to overestimation of treatment effects; however, consistent findings across trials, geographic regions, and patient subgroups reinforce the efficacy of EVT in extensive ischemic strokes.

## Conclusion

In AIS patients with LVO-related large ischemic infarcts, EVT and MC yielded statistically significant long-term functional improvements compared with MC alone. EVT’s benefits increased with longer follow-up and were most pronounced in patients with shorter onset times and smaller infarct volumes.

## Data Availability

The data used in this meta-analysis are derived from published randomized controlled trials (RCTs) that are publicly available in electronic databases including PubMed/MEDLINE, Scopus, and Web of Science. The full texts of the included RCTs can be accessed via the official websites of these databases with appropriate subscriptions. The detailed list of included studies, including their DOI numbers and publication information, is available from the corresponding author upon reasonable request.
