# Supplementary material for: Long-term outcomes of endovascular thrombectomy vs. medical care in patients with large ischemic stroke: a systematic review and meta-analysis of randomized controlled trials
Source: Front Neurol. 2026 May 13;17:1776595. doi: 10.3389/fneur.2026.1776595 (PMC13215127; doi:10.3389/fneur.2026.1776595)
Supplement: Supplementary file 1 [file Table_1.DOCX]

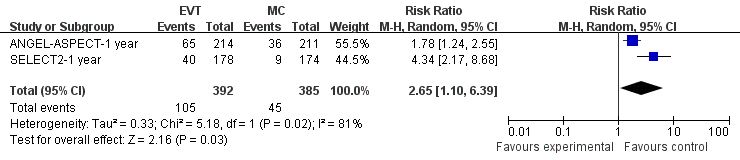


Fig 5. Forest plot of meta-analysis for functional independence (mRS 0–2) in patients selected by perfusion imaging.


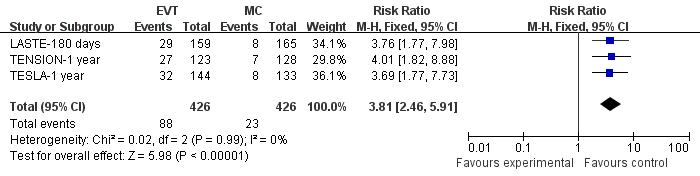


Fig 6. Forest plot of meta-analysis for functional independence (mRS 0–2) in patients selected by no-perfusion imaging.


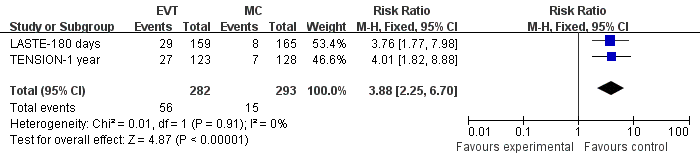


Fig 7. Forest plot of meta-analysis for functional independence (mRS 0–2) in patients selected by presented≤12h.


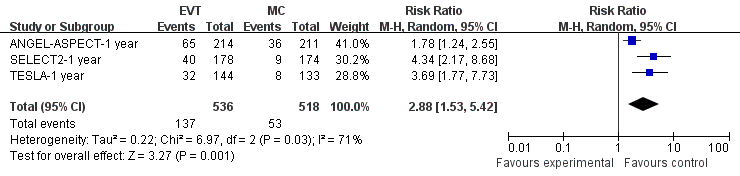


Fig 8. Forest plot of meta-analysis for functional independence (mRS 0–2) in patients selected by presented≤24h.


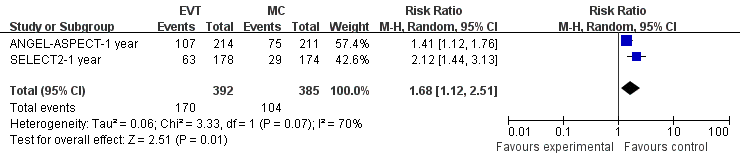


Fig 9. Forest plot of meta-analysis for functional independence (mRS 0–3) in patients selected by perfusion imaging.


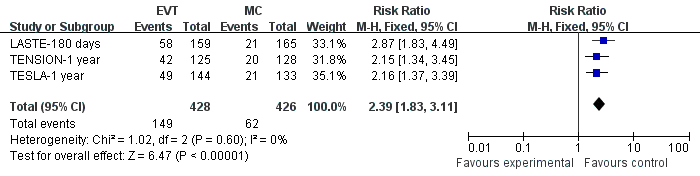


Fig 10. Forest plot of meta-analysis for functional independence (mRS 0–3) in patients selected by no-perfusion imaging.


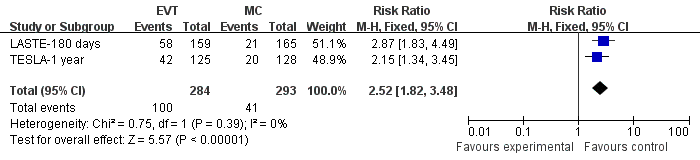


Fig 11. Forest plot of meta-analysis for functional independence (mRS 0–3) in patients selected by presented≤12h.


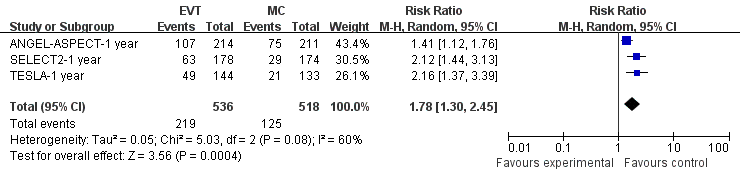


Fig 12. Forest plot of meta-analysis for functional independence (mRS 0–3) in patients selected by presented≤24h.


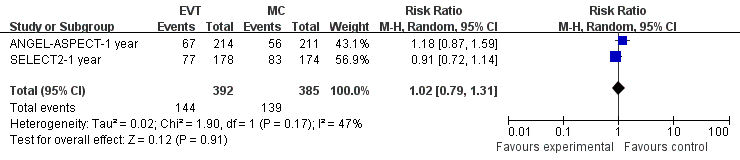


Fig 13. Forest plot of meta-analysis for mortality in patients selected by perfusion imaging.


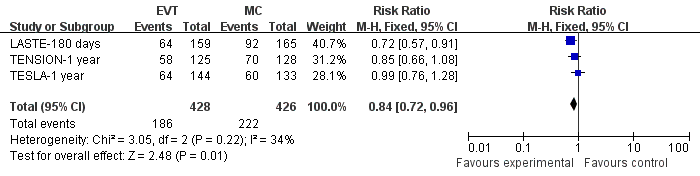


Fig 14. Forest plot of meta-analysis for mortality in patients selected by no-perfusion imaging.


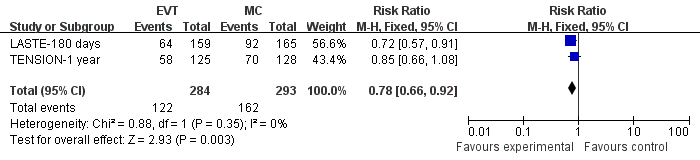


Fig 15. Forest plot of meta-analysis for mortality in patients selected by presented≤12h.


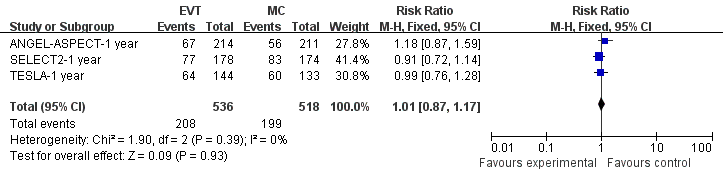


Fig 16. Forest plot of meta-analysis for mortality in patients selected by presented≤24h.


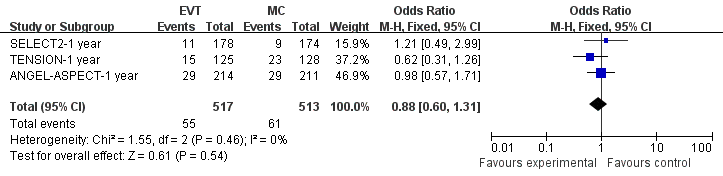


Fig 17. Forest plot of 1‑year mRS shift in patients with ASPECTS 0–2.


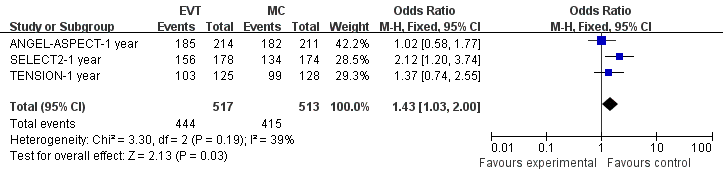


Fig 18. Forest plot of 1‑year mRS shift in patients with ASPECTS 3–5.


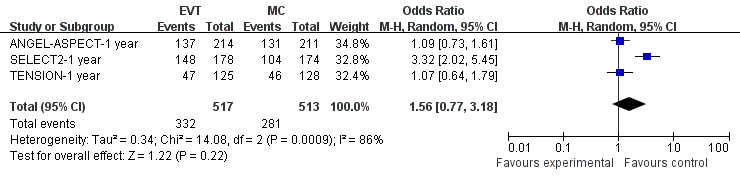


Fig 19. Forest plot of 1‑year mRS shift in patients with present ≥6h.


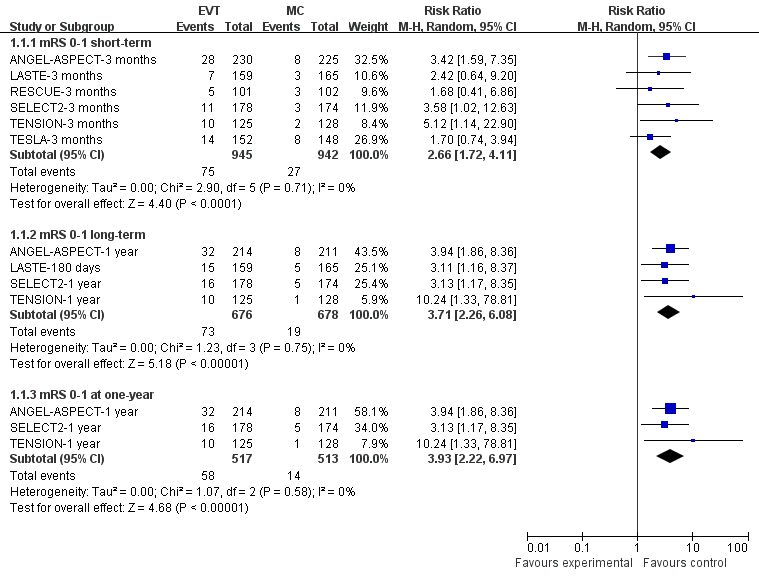


Fig 20. Sensitivity analysis by changing the analytical model for functional excellence (mRS 0–1).


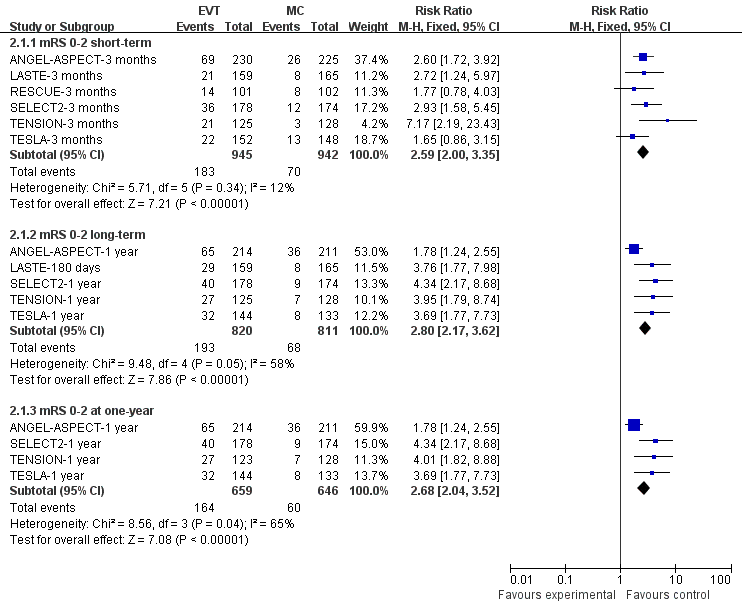


Fig 21 Sensitivity analysis by changing the analytical model for functional independence (mRS 0-2).


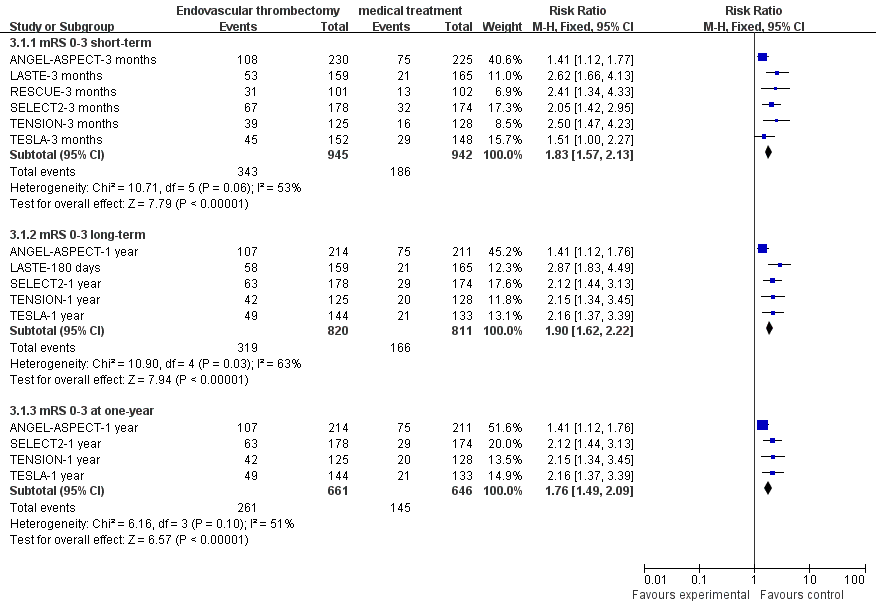


Fig 22 Sensitivity analysis by changing the analytical model for Independent Ambulation (mRS 0-3).


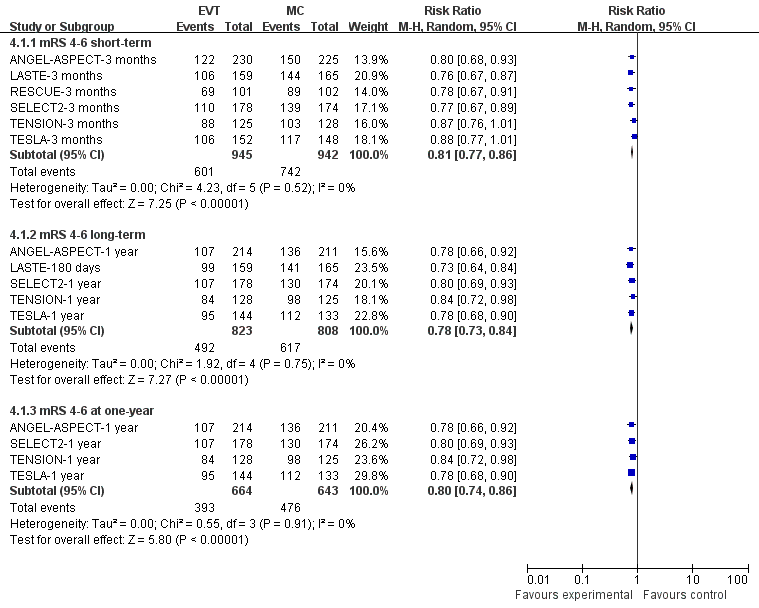


Fig 23 Sensitivity analysis by changing the analytical model for Death or Dependency (mRS 4-6).


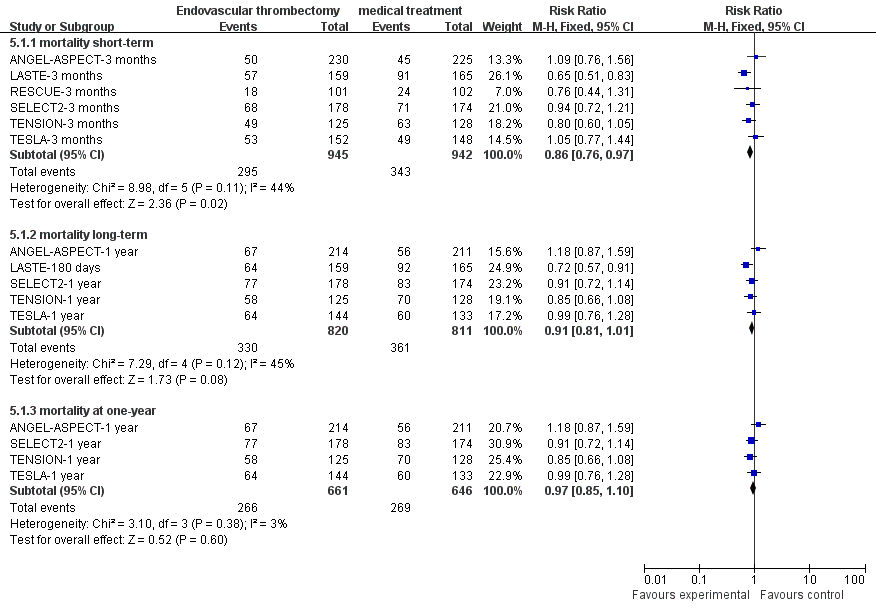


Fig 24 Sensitivity analysis by changing the analytical model for mortality.
